# Extracellular Vesicles Derived From Canine Mesenchymal Stromal Cells in Serum Free Culture Medium Have Anti-inflammatory Effect on Microglial Cells

**DOI:** 10.3389/fvets.2021.633426

**Published:** 2021-04-28

**Authors:** Yukina Kuwahara, Karin Yoshizaki, Hidetaka Nishida, Hiroaki Kamishina, Sadatoshi Maeda, Katsura Takano, Naoki Fujita, Ryohei Nishimura, Jun-ichiro Jo, Yasuhiko Tabata, Hideo Akiyoshi

**Affiliations:** ^1^Joint Department of Veterinary Medicine, The United Graduate School of Veterinary Sciences, Gifu University, Gifu, Japan; ^2^Department of Veterinary Surgery, Graduate School of Life and Environmental Sciences, Osaka Prefecture University, Izumisano, Japan; ^3^Department of Integrative Physiology, Graduate School of Life and Environmental Sciences, Osaka Prefecture University, Izumisano, Japan; ^4^Department of Veterinary Surgery, Graduate School of Agricultural and Life Sciences, The University of Tokyo, Tokyo, Japan; ^5^Laboratory of Biomaterials, Institute for Frontier Life and Medical Sciences, Kyoto University, Kyoto, Japan

**Keywords:** exosome, extracellular vesicles, canine, mesenchymal stromal cells, anti-inflammatory, serum free culture

## Abstract

Mesenchymal stem/stromal cells (MSCs) have been used as cell sources for treating dogs with naturally-occurring diseases. Extracellular vesicles (EVs) derived from MSCs are now recognized as pivotal to modulating the immune response and supporting tissue repair. Manufacture of MSC-EVs for clinical application mandates removal of the xeno-proteins, including fetal bovine serum. The objective of this study was to examine whether canine MSCs survived and secreted EVs in serum-free medium (SFM) conditions and to assess the immunomodulatory effect of EVs *in vitro*. Canine MSCs were found to survive and secrete EVs under SFM conditions. The surface markers of MSCs in the SFM were similar to MSCs in complete culture medium. Canine MSC-EVs had a diameter of ~300 nm and were positive for EV markers. MSC-derived EVs from the serum-free condition reduced the levels of *IL-1*β by BV-2 cells in response to LPS stimulation. These results warrant further studies of the use of SFM for producing EVs derived from canine MSCs.

## Introduction

Mesenchymal stem/stromal cells (MSCs) are one of the most commonly utilized biologics in the field. MSCs can be easily isolated from various connective tissue including bone marrow, blood and fat. MSCs have the capacity to differentiate into osteoblasts, adipocytes and chondrocytes (1, 2). The therapeutic effect of MSCs was initially thought to be derived from the ability of these cells to differentiate into multiple cell types (2). However, it was frequently observed that therapeutic effect after administration of MSCs does not correlate with engraftment or differentiation of MSCs (3, 4). These observations led to the view that MSCs suppress the function of various immune effector cells and support tissue repair through secretion of various immune modulatory factors (5–8).

Several studies (3, 9, 10) using rodent models have shown that injection of MSCs promotes functional recovery from traumatic brain injury and spinal cord injury (SCI). Some transplanted MSCs attached themselves to the spinal cord near the surface, with a few invading the lesion (3, 11). Injection of MSCs modified the inflammatory environment and reduced the size of the injury site and increased myelin sparing (12). The clinical use of intrathecal injection of MSCs to canine patients with severe SCI has been reported by our research group (13).

There are limitations in clinical applications using stem cell therapy in dogs and humans, however. Injection of MSCs carries inherent risk, including occlusion in microvasculature and transformation of MSCs into inappropriate cell type and cancer (14, 15). The cell-free therapies resolve several safety issues associated with the transplantation of living cells. Several studies using rodent models have shown that MSC-conditioned media repairs tissue injury (16, 17). The therapeutic effects of MSCs are now recognized to be mediated by various cytokines, growth factors, and extracellular vesicles (EVs) (18–20).

EVs are composed of lipid bilayers that are produced by various cell types. These are classified into three main classes, exosomes, microvesicles, and apoptotic bodies, based on the mode of biogenesis. Exosomes are formed as the intraluminal vesicles of multivesicular bodies during the maturation of endosomes, and are secreted from cells by exocytosis after the fusion of multivesicular bodies with a plasma membrane (21). Microvesicles and apoptotic bodies bud directly from the plasma membrane (22). As standards have not been established to separate or classify the different types of vesicles, the collective term “extracellular vesicles” is recommended. EVs encapsulate mRNA, miRNA and proteins, and act in cell-cell communication by shuttling complex messages. MSC-EVs, are easily preserved by freezing allowing them to be rapidly available for therapeutic use. EVs can be stored without any potentially toxic cryopreservative agents for a long period. EVs can be transferred to targeted recipient cells, including neurons (23) and microglial cells (24). There have been reports that EVs derived from MSCs reduced immune response and promoted tissue repair in rodents (9). Several clinical studies (25, 26) have been published in which MSC-EVs were applied to human patients. There is little knowledge about EVs derived from canine MSCs.

The Food and Drug Administration requires that MSC products conform to good manufacturing practice, and encourages the use of xeno-protein free culture conditions, including fetal bovine serum (FBS). FBS contains many proteins, growth factors and EVs derived from xenogeneic animals. A culture condition without FBS is therefore needed in the development of therapies using MSC-derived EVs. The purpose of this study was to assess whether canine MSCs survive and secrete EVs in serum-free media (SFM). A second goal was to study the immunomodulatory effect of EVs *in vitro*.

## Materials and Methods

### Experimental Animals

Five healthy beagles of age 1–4 years (median age, 2 years) were used in the study. The dogs belonged the kennel of Research Center for Experimental Animal Science of Osaka Prefecture University. The dogs were considered healthy based on their medical history and physical examination. All procedures were performed in accordance with the guidelines of the Experimental Animal Committee of Osaka Prefecture University and the protocol was approved by the Experimental Animal Committee of Osaka Prefecture University (Approval Number 30-181).

### Isolation and Culture of Canine MSCs

Canine MSCs were isolated from five healthy beagles and were cultured as described previously (27). The bone marrow perfusate was centrifuged and the precipitates were suspended in 15 mL Dulbecco PBS solution (Nakalai Tesque, Kyoto, Japan). The mononuclear cells were isolated by density centrifuging with a lymphocyte separation solution (Nakalai Tesque, Kyoto, Japan) at 400 × g for 30 min at room temperature. The buffy coat at the interface was collected, mixed with 20 mL Dulbecco PBS solution and centrifuged at 300 × g for 5 min. The precipitated cells were washed with Dulbecco PBS solution. The number of cells was determined using a hemacytometer. Enriched mononuclear cells were plated in 15 cm tissue culture dishes (Corning, Rochester, NY) at a density of 1.5 × 10^5^ cells/cm^2^ in complete culture medium, consisting of Dulbecco modified eagle medium (DMEM low glucose) (Nakalai Tesque, Kyoto, Japan) with 10% heat-inactivated fetal bovine serum (FBS) (GE healthcare, Chicago, IL) and 1% antibiotic-antimycotic solution (Nakalai Tesque, Kyoto, Japan), and incubated at 37°C in 5% CO_2_. Non-adherent cells were removed by replacing the medium 48 h after plating. The culture medium was changed twice per week. Upon reaching 80% confluence, 0.25% trypsin–EDTA solution (Nakalai Tesque, Kyoto, Japan) was used to harvest the adherent cells. The collected cells were centrifuged at 300 × g for 5 min, washed with PBS solution, and then cryopreserved in liquid nitrogen. These cells were termed passage 1.

Frozen MSCs from five healthy beagles were thawed at 37°C and plated directly in tissue culture dishes of diameter 10 cm (Corning, Rochester, NY). After 24 h, MSCs were seeded at 6.0 × 10^4^ cells per cm^2^ (passage 2). After the cells reached about 70–80% confluency, the culture plates were washed with PBS solution, and each dish was replaced with 15 mL of CCM containing EV depleted FBS (CCM+FBS), CCM without FBS (CCM–FBS), and two kinds of SFM: SFM 1 (MSC NutriStem XF Medium: Biological Industries, Beit Haemek, Israel) or SFM 2 (STEMPRO MSC SFM: Thermo Scientific, Carlsbad, CA), respectively. CCM+FBS was ultracentrifuged (Himac CS 120GX2 Micro Ultracentrifuge and S52ST Rotor: HITACHI, Tokyo, Japan) at 100,000 × g for 12 h at 4°C for depletion of EVs derived from FBS.

### Live and Dead Cell Staining

The proportion of apoptotic cells was assessed using a commercials available kit (Annexin V-FITC Apoptosis Detection Kit: Nakalai Tesque, Kyoto, Japan) according to the manufacturer's instructions. MSCs were suspended with annexin-V Binding Buffer at a concentration of 1.0 × 10^6^ cells/ml and 100 μl of cell suspension was transferred to another tube. Cells were stained with 5 μl of FITC-labeled annexin-V and 5 μl of propidium iodide (PI) solution for 15 min in the dark at room temperature. The proportion of apoptotic MSCs was quantified using a flow cytometer (BD FACSCANTO 2: BD bioscience, Tokyo, Japan). Data were analyzed by recording 10,000 events. Individual cells were characterized on the basis of forward scatter (related to cell size) and orthogonal scatter (related to cell granularity). Living non-apoptotic cells yielded negative results for FITC-labeled annexin-V and PI. Cells that gave positive results for FITC-labeled annexin-V and negative results for PI were considered apoptotic, and cells that had positive results for FITC-labeled annexin-V and PI were considered necrotic or dead. The survival rate was calculated from the population of living non-apoptotic cells per total cell.

### Surface Markers of MSCs

For assay of cell surface markers canine MSCs from three healthy beagles were cultured in CCM+FBS, SFM1, and SFM2, a flow cytometric analysis was performed. Cells (passage 2) were detached from the dishes with 0.25% trypsin-EDTA and were collected by centrifuging. Aliquots containing 1.0 × 10^6^ cells were washed in PBS solution containing 2.0% FBS. They were then incubated in 100 μl of fluorescence-activated cell sorting buffer with fluorescent isothiocyanate and phycoerythrin-labeled antibodies for mouse monoclonal anti-canine CD34 (clone: 1H6, Thermo Scientific, Carlsbad, CA) at 0.25 μg/mL (1:200), rat monoclonal anti-canine CD44 (clone: YKIX337.8, Thermo Scientific, Carlsbad, CA) at 0.25 μg/mL (1:100), rat monoclonal anti-canine CD45 (clone: YKIX716.13, Thermo Scientific, Carlsbad, CA) at 0.25 μg/mL (1:200), and rat monoclonal anti-canine CD90 (clone: YKIX337.217, Thermo Scientific, Carlsbad, CA) at 0.25 μg/mL (1:200) for 20 min at room temperature. In a negative control sample, isotype control was used (Mouse IgG1 kappa isotype Control-PE: Thermo Scientific, Carlsbad, CA, Rat IgG2a kappa Isotype Control-FITC: Thermo Scientific, Carlsbad, CA, Rat IgG2b kappa Isotype Control-FITC: Thermo Scientific, Carlsbad, CA, Rat IgG2b kappa Isotype Control–PE: Thermo Scientific, Carlsbad, CA). Data were analyzed by recording 10,000 events on by the flow cytometer (CytoFlex S: Beckman Coulter, Brea, CA). The data were analyzed using CytoExpert (Beckman Coulter, Brea, CA).

### Isolation of EVs by Ultracentrifugation

Frozen MSCs from five healthy beagles were thawed at 37°C and plated directly in tissue culture dishes of diameter 10 cm (Corning, Rochester, NY). After the cells (passage 2) had reached about 70–80% confluency, the culture plates were washed with PBS solution, and each dish was replaced with CCM+FBS and two types of SFM. After 48 h, the culture supernatant was collected from each dish, for isolation of EVs. The culture supernatant was centrifuged at 2,320 × g for 15 min to remove cells and debris, and was then ultracentrifuged at 100,000 × g, for 90 min at 4°C. The resulting pellet was resuspended in PBS solution. The protein content was quantified by the Bradford method (Nakalai Tesque, Kyoto, Japan).

### Western Blotting

Equal volume of samples were heat blocked in SDS-PAGE sample buffer under reducing conditions, and denatured at 100°C for 5 min. The samples were electrophoresed on 10% SDS polyacrylamide gels, and transferred on to 0.45 μm nitrocellulose membrane (Bio-Rad, Hercules, CA). The membrane was incubated for 2 h in 3% blocking buffer (ECL Prime^TM^ blocking agent: GE Healthcare, Chicago, IL) and probed with mouse monoclonal anti-TSG101 antibody (clone: 51/TSG101, BD bioscience, Tokyo, Japan) at 1:500 at 4°C overnight. The membrane was washed and incubated with HRP-conjugated goat anti-mouse antibody (Peroxidase AffiniPure Goat Anti-Mouse IgG: Jackson ImmunoResearch Inc., West Grove, PA) at 1:2000 for 1 h at room temperature. Protein was detected using chemiluminescent substrate (LAS4000: FUJIFILM, Tokyo, Japan).

### Assay of EVs With Particle Size Distribution Analysis

The particle size of MSC-derived EVs from CCM+FBS, SFM1, and SFM2 was measured *via* dynamic light scattering (Zetasizer Nano ZS: Malvern, Worcestershire, UK). The analysis was performed at 25°C, using samples diluted with PBS solution.

### EV Uptake Into BV-2 Microglia Cells

EVs derived from CCM+FBS, SFM1, and SFM2 were labeled with PKH26 (Sigma, St. Louis, MO), as described previously (28). BV-2 cells were cultured in DMEM with 5% heat-inactivated FBS and 1% antibiotic-antimycotic solution at 37°C in 5% CO_2_. After BV-2 cells reached about 70–80% confluency, they were detached using 0.25% trypsin-EDTA solution and seeded at 2.5 × 10^5^ cells per chamber in chamber slides (Iwaki, Tokyo, Japan) overnight. The labeled EVs were added and co-cultured for 6 h. The nuclei of BV-2 cells were stained with 4′,6-diamidino-2′-phenylindole-dihydrochloride (DAPI) (Invitrogen, Carlsbad, CA). Fluorescence microscope images were acquired using a Virtual Slide System (Olympus Corporation, Tokyo, Japan).

### BV-2 Microglia Cell Stimulation and Co-culture With MSC-EVs

BV-2 cells were cultured in DMEM with 5% heat-inactivated FBS and 1% antibiotic-antimycotic solution at 37°C in 5% CO_2_. After BV-2 cells reached about 70–80% confluency, they were detached using 0.25% trypsin-EDTA solution and seeded at 5.0 × 10^5^ cells per well in a 6-well plate. After 24 h of incubation, BV-2 cells were stimulated by replacing 1 mL of cultured medium alone, or cultured medium containing 1 ng/mL of lipopolysaccharide (LPS) (Sigma, St. Louis, MO), LPS in combination with MSC-derived EVs. MSC-derived EVs were prepared from the conditioned media that 1 × 10^6^ MSCs were cultured for 48 h, and added in each well. Each assay was tested using MSC-derived EVs collected from different five healthy beagles. The levels of gene expression in non-LPS treated BV-2 cells were expressed as 1 U.

### Real-Time Quantitative PCR

Total RNA was extracted using a NucleoSpin RNA Plus (MACHEREY-NAGEL, Nordrhein-Westfalen, Netherlands) according to the manufacturer's instructions. The RNAs were converted to cDNA with reverse transcriptase using oligo (dT) primers to prime ReverTraAce (TOYOBO, Osaka, Japan). The cDNA was then amplified by the SYBR Green Realtime PCR Master-Mix-Plus (TOYOBO, Osaka, Japan) using a StepOnePlus Real-Time PCR system (Applied Biosystems, Foster City, CA). The following primers were used in this study:5′-TCCAGGATGAGGACATGAGCAC-3′ (forward) and 5′-GAACGTCACACA CCAGCAGGTTA-3′ (reverse) for *IL-1*β; 5′-ATGAGCACAGAAAGCATGATC-3′ (forward) and 5′- TACAGGCTTGTCACTCGAATT-3′ (reverse) for *TNF-*α; 5′-ACACATGTTCTTCTGGGAAATCG-3′ (forward) and 5′-TGAAGGACTCTGGCTTTGTC-3′ (reverse) for *IL-6*; and 5′-CACTCACGGCAAATTCAACGGCAC-3′ (forward) and 5′-GACTCCACGACATACTCAGCAC-3′ (reverse) for *GAPDH*. The optimum conditions for PCR amplification of the cDNA were established according to the manufacturer's instructions. Reactions were carried out as follows: initial denaturation at 95°C for 30 s, followed by 40 cycles of 5 s at 95°C and 30 s at 60°C. The data were analyzed using StepOne software (Applied Biosystems, Foster City, CA), and the cycle number at the linear amplification threshold (Ct) values for the endogenous control gene (*GAPDH*) and the target gene were recorded. The relative gene expression was calculated using the comparative Ct method (2^−Δ*ΔCt*^). The levels of gene expression in non-LPS treated BV-2 cells were expressed as 1 U.

### Statistical Analysis

Data are presented as mean ± SD. The percentages of apoptotic cells were performed *via* one-way analysis of variance and a Bonferroni post-test using StatView 5.0 (SAS Institute Inc., Cary, NC). Real-time quantitative PCR were performed by Kruskal-Wallis-test followed by Dunn's multiple-test using GraphPad prism 7 software (GraphPad Software Inc., San Diego, CA). Values of *p* < 0.05 were taken as significant.

## Results

### Identification of Canine MSCs

After reaching 80% confluence, the cells were collected. Expression of surface markers of attached cells were CD44 and CD90 positive and negative for CD34 and CD45. These surface markers are consistent with phenotypes observed in canine MSCs (25). According to these characteristics, these cells conform to canine MSC.

### Survival Rates of Canine MSCs Cultured in Serum-Free Condition

**Figure 1:**
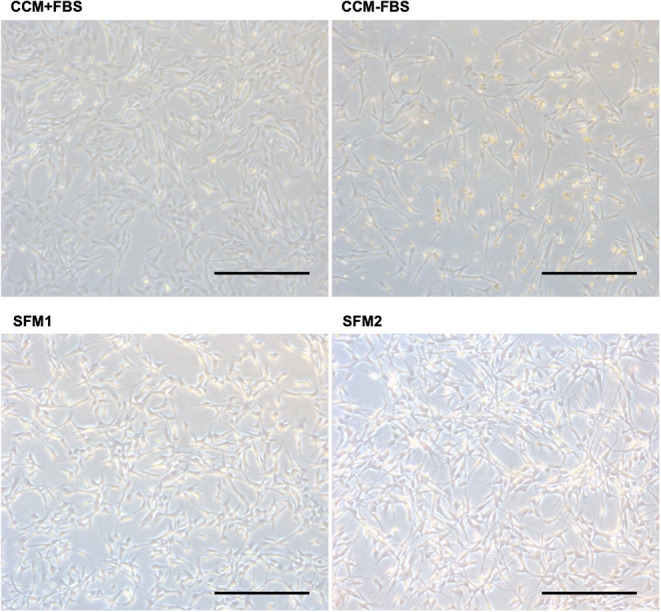
Photomicrographs of canine MSCs. Representative photomicrographs of canine MSCs cultured in CCM+FBS, CCM–FBS and two kinds of SFM; SFM 1 and SFM 2. Canine MSCs were dead and floated in the CCM–FBS. Bars = 400 μm.

**Figure 2:**
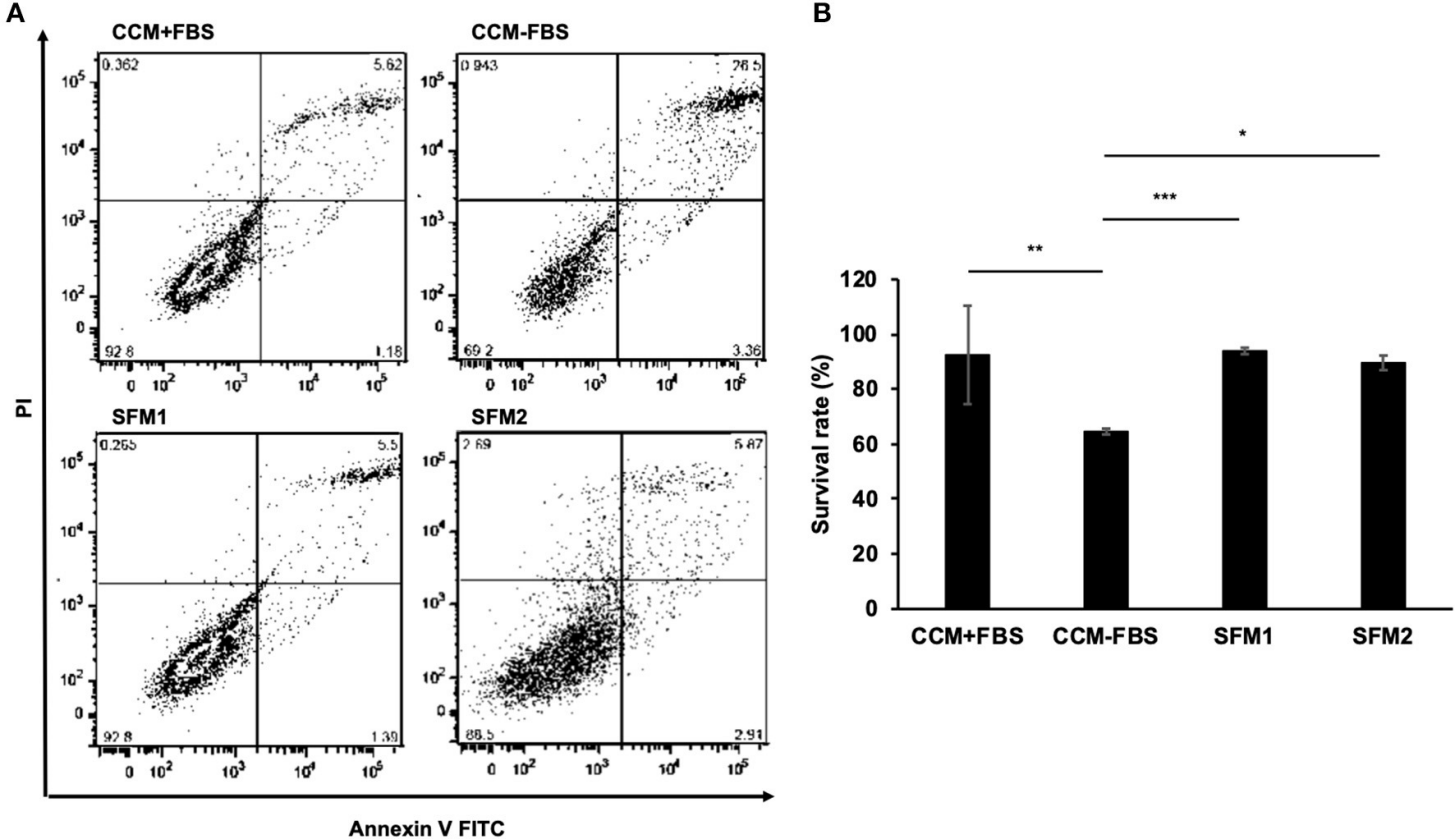
Survival rates of canine MSCs. **(A)** Representative dot plots of Annexin V FITC-PI double staining with canine MSCs cultured in CCM+FBS, CCM–FBS, two kinds of SFM: SFM 1 and SFM 2. **(B)** Mean ± SD percentage of viable cells cultured in CCM+FBS, CCM–FBS, SFM 1 and SFM 2. Survival rates were calculated from the number of viable cells divided by total cell counts. There was no significant difference between the survival rate of MSCs in CCM+FBS and SFM (SFM 1 and SFM 2). The proportion of surviving cells in CCM+FBS, SFM 1 and SFM 2 was significantly higher than that of CCM–FBS (*p < 0.05, **p < 0.01, ***p < 0.001).

When culturing canine MSCs using CCM+FBS, MSCs survived and grew (Figure 1). When culturing canine MSC using CCM–FBS, 35% of cells were dead and floated. Canine MSCs survived and grew in the SFM 1, 2 which did not contain FBS. The cells were triangular or star-like shaped in CCM or SFM-1. The cells were spindle shaped after the change of SFM 2. Canine MSCs cultured in the two SFMs had similar survival rates to that of canine MSCs cultured in CCM+FBS (CCM+FBS: mean 92.50 ± 17.91%, CCM–FBS: 64.60 ± 1.08%, SFM 1: mean 93.95 ± 1.19%, SFM 2: mean 89.62 ± 2.66%) (Figure 2A). The proportion of surviving cells in CCM+FBS, SFM 1 and SFM 2 was significantly higher than that for CCM–FBS (CCM+FBS: *p* < 0.01, SFM1: *p* < 0.001, SFM 2: *p* < 0.05). There was no significant difference between the survival rates of MSCs in SFM (SFM 1 and SFM 2) and CCM+FBS (Figure 2B).

### Phenotypic Analysis of Cell Surface Markers of Canine MSCs in Serum-Free Condition

**Figure 3:**
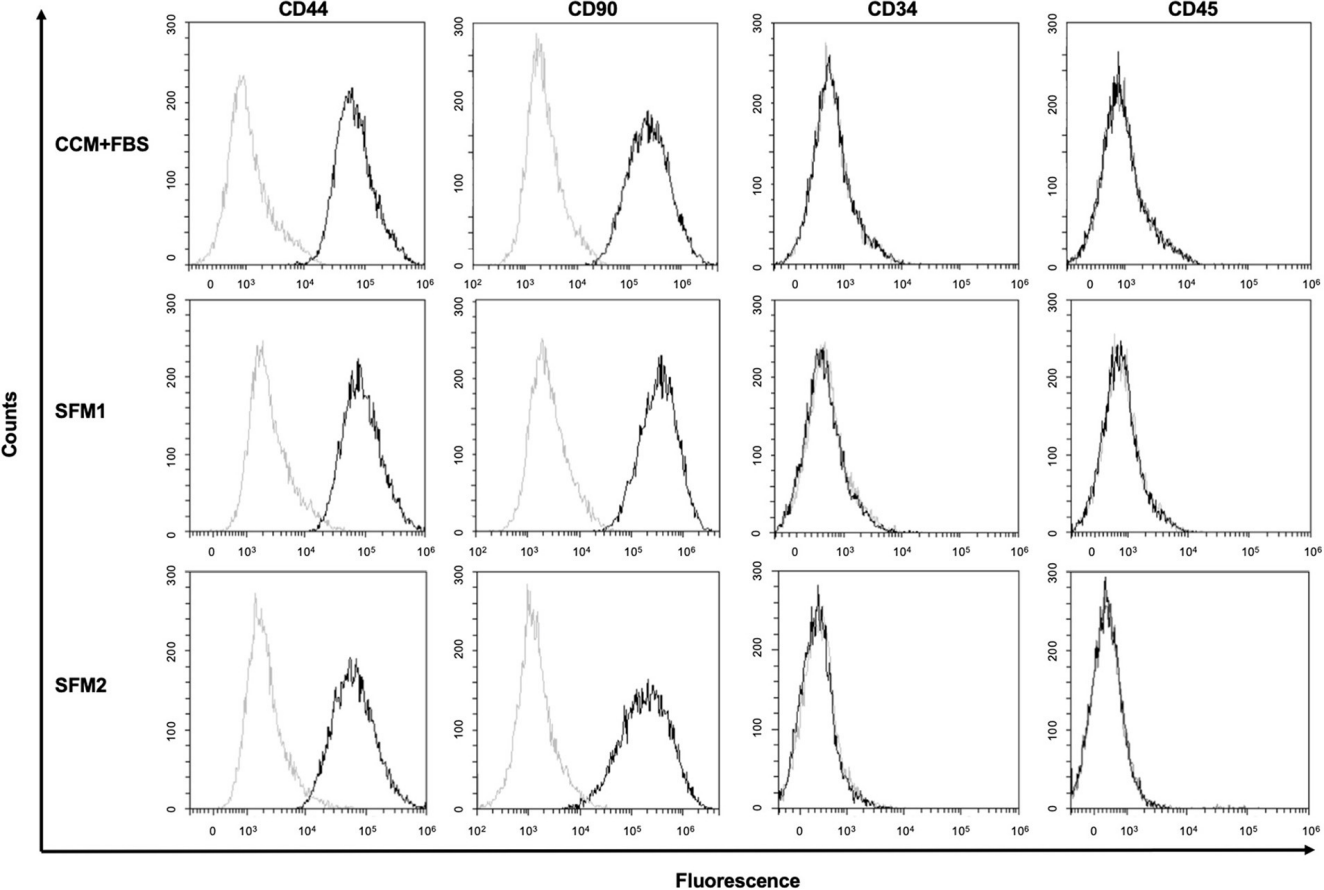
Surface marker of canine MSCs. Representative results of flow cytometric analysis of canine MSCs derived from CCM+FBS, SFM 1 and SFM 2 (black line). Isotype controls are shown in each panel (gray line).

CCM+FBS, SFM1, and SFM2 showed similar expression of surface antigens: CD34 (CCM+FBS: 0.45 ± 0.35%, SFM1: 0.29 ± 0.24%, SFM2: 0.05 ± 0.03%), CD44 (CCM+FBS: 97.74 ± 1.20%, SFM1: 96.69 ± 2.22%, SFM2: 96.42 ± 2.28%), CD45 (CCM+FBS: 0.10 ± 0.13%, SFM1: 0.14 ± 0.19%, SFM2: 0.02 ± 0.02%), and CD90 (CCM+FBS: 97.91 ± 1.04%, SFM1: 98.62 ± 0.77%, SFM2: 96.66 ± 0.39%) (Figure 3). There was no difference of the surface antigens between groups.

### Isolation of MSC-Derived EVs by Ultracentrifugation

**Figure 4:**
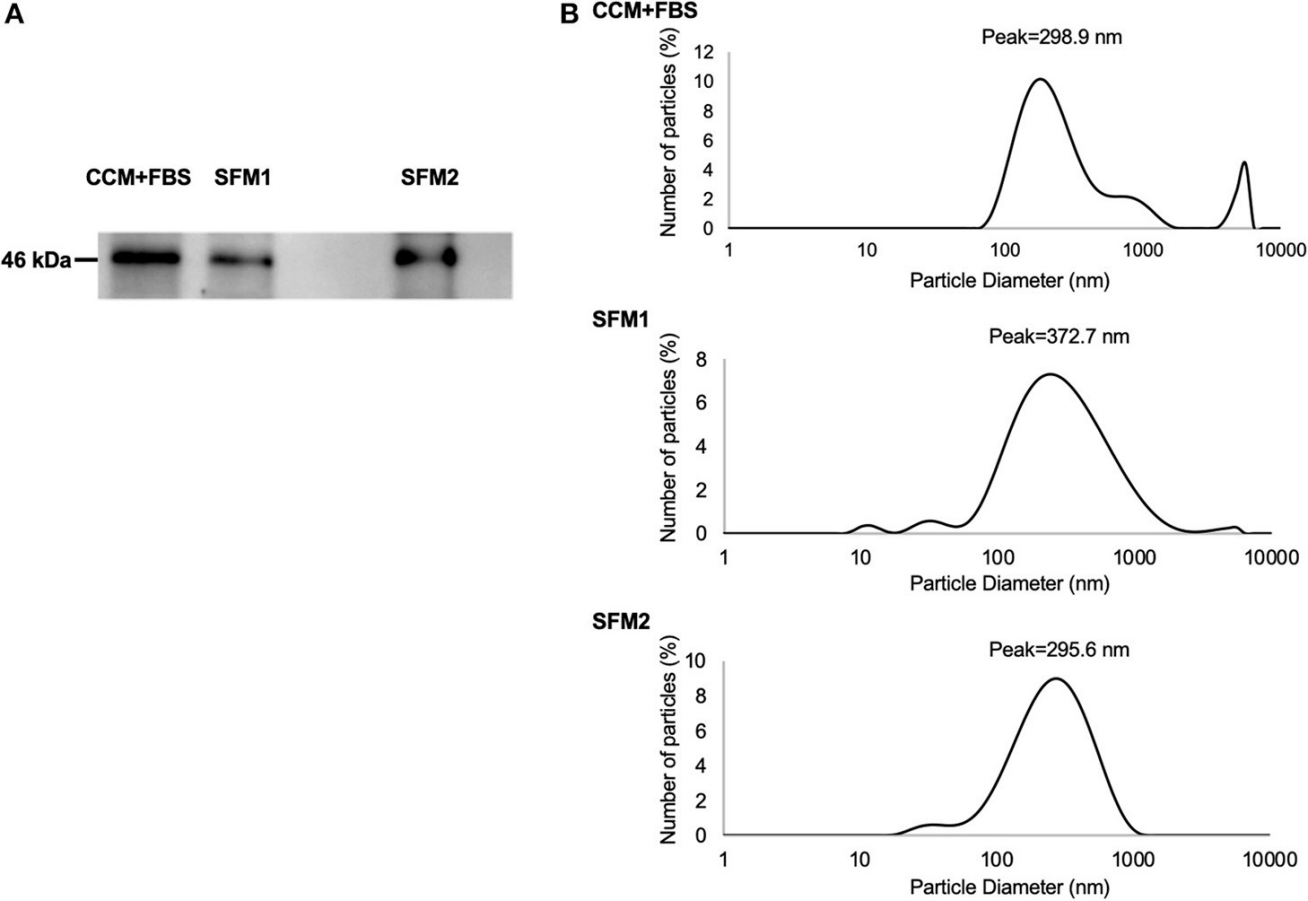
Characteristics of MSC-derived EVs. **(A)** Expression of TSG101 of MSC-derived EVs from CCM+FBS, SFM 1 and SFM 2. **(B)** Diameter of MSC-derived EVs from CCM+FBS, SFM 1 and SFM 2 with scattering intensity peaks at about 298.9, 372.7, and 295.6 nm.

Expression of TSG101 was detected in MSC-derived EVs from CCM+FBS, SFM 1 and SFM 2 (Figure 4A). Expression of TSG101 was observed in MSC-derived EVs from five dogs (data not shown). To determine the size distribution of EVs isolated from MSCs (*n* = 1 dog), a dynamic light scattering was performed. The particle size of the EVs of canine MSC was measured using a zetasizer. The diameter of EVs derived from canine MSCs cultured in CCM+FBS, SFM 1 and SFM 2 showed scattering intensity peaks at about 298.9, 372.7, and 295.6 nm (Figure 4B).

### Immunomodulatory Effect of MSC-Derived EVs

**Figure 5:**
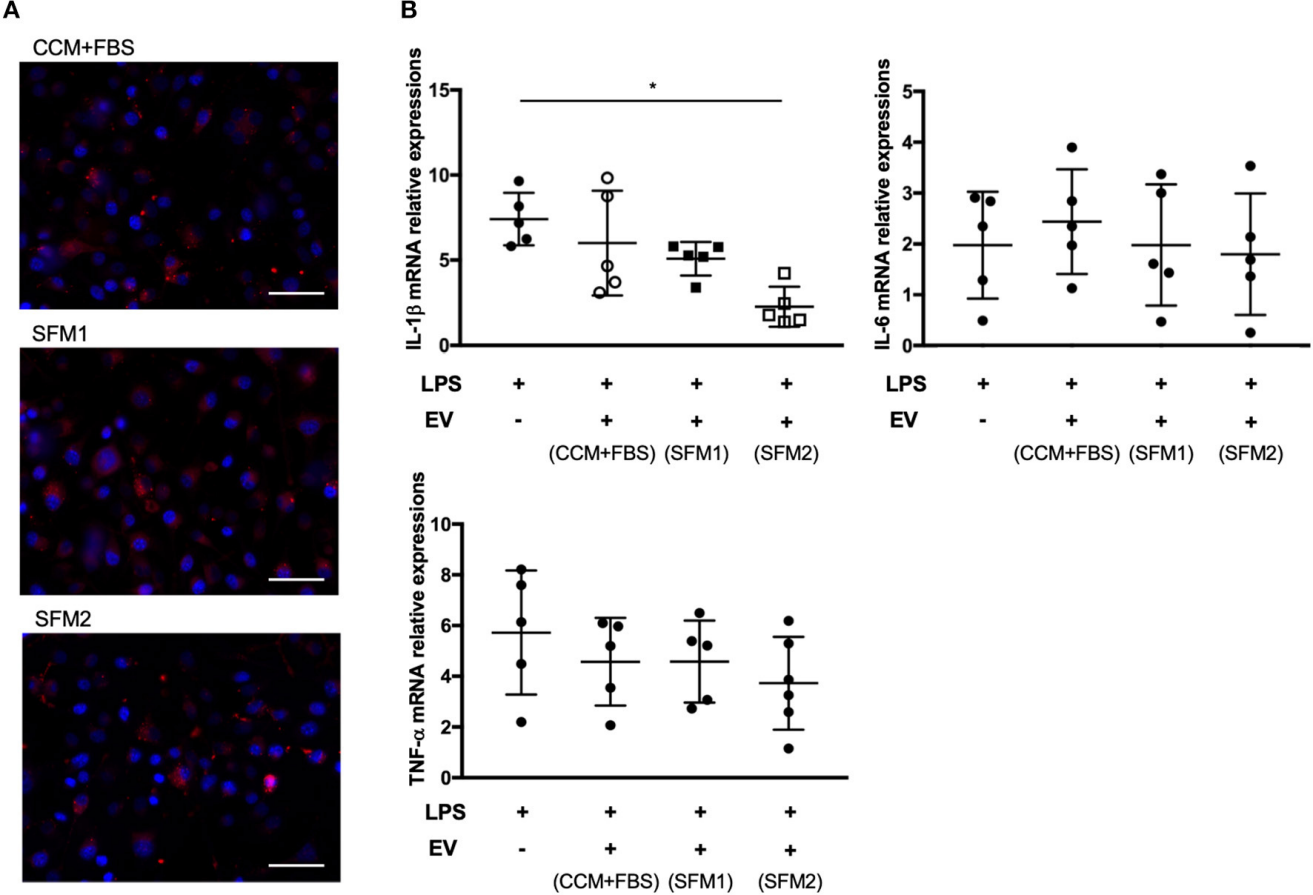
Anti-inflammatory effect of MSC-derived EVs on BV-2 microglia cells. **(A)** Representative fluorescence microscope image showing the internalization of PKH26-labeled EVs (red) by BV-2 microglial cells. Bars = 50.0 μm **(B)** qRT-PCR assay for the expression of *IL-1*β, *TNF-*α, and *IL-6* in BV-2 microglial cells incubated with lipopolysaccharide (LPS) and canine MSC-derived EVs from CCM+FBS, SFM 1 and SFM 2. Individual PCR reactions were normalized against the internal control *GAPDH*. (*p < 0.05) The levels of gene expression in non-LPS treated BV-2 cells were expressed as 1 U.

Canine MSC-derived EVs derived from CCM+FBS, SFM 1 and 2 were added to cultures of BV-2 cells (Figure 5A). The expression of *IL-1*β in LPS-stimulated BV-2 cells was lower in group treated with EVs derived from SFM2 than untreated group (Figure 5B). There was no significant difference between groups in levels of *TNF-*α and *IL-6*.

## Discussion

To develop cell-free therapy using canine MSC-EVs, we assessed whether canine MSCs survived and secreted EVs in SFM conditions. It has been reported that human MSCs can survive even with basal medium not containing FBS (29). Our results, however, indicate that about 35% of canine MSCs were dead and floated in the medium without FBS. Human MSCs have proliferative capacities in basal medium not containing FBS, but there are also reports of induced senescence *in vitro* (30). The conditions necessary for culturing canine MSCs might be different from humans. In previous reports (29), SFM 2 promoted an increase of expression of anti-inflammatory cytokines. Canine MSC-EVs from CCM+FBS have not been shown to reduce the expression of *IL-1*β, but canine MSC-EVs from SFM 2 significantly decreased the expression of *IL-1*β. Many culture conditions, including pH, O_2_ tension, culture medium, mechanical cues, and inflammatory stimuli, can influence MSC phenotype and properties (31). Several studies indicated the secretome of MSCs can be modulated by the culture condition (32, 33). Consistent with our observations, human MSC-derived EVs also reduced the LPS-induced expression of inflammation-related genes by BV-2 cells (24). Pacienza et al. have also developed an assay for the anti-inflammatory activity of EVs with a line of mouse macrophage (34). They develop an *in vitro* model that correlates with an *in vivo* model (34). Our results suggest that SFM 2 conditions could modulate secretion profile and be useful for clinical applications.

The diameter of EVs in SFM 1 gave larger scattering intensity peaks than in SFM 2. Patton et al. found that changes in the size distribution of EVs took place under extreme hypoxia (35). The difference in size distribution of EVs in SFM 1 and SFM 2 may reflect the culture condition of canine MSCs. One limitation of the study was that MSCs from one a single donor were utilized to determine the size of EVs. In previous reports, the removal of FBS from canine MSC culture systems led to altered immunomodulatory properties (29). EVs derived from canine MSCs appear to vary according to certain biological conditions (36). The molecules, including TSG101, Rab-5b, CD9, CD63, and CD81, are thought be essential components of EVs. Yoshioka et al. reported that some EV markers, including TSG101, Rab-5b, and CD63, were detected in EVs, but their abundance varied according to their origin (37). The other EV molecules such as CD9 and CD81 could be useful as marker proteins for the quantitative analyses of EV proteins. For the BV-2 assay, we used the same numbers of MSCs to compare the immunomodulatory effects of EVs, and the number of EVs derived from each medium were not measured. It was not clear whether the difference of immunomodulatory effect was due to the amount or properties of EVs. Further studies are needed to compare the characterization of EVs derived from different culture condition.

The cells were triangular or star-like shaped in CCM or SFM-1. The morphology of canine MSCs was spindle shaped following SFM 2 condition. The cell morphology depends on cytoskeleton organization, cell adhesion and activated pathways (38). Katsube et al. reported that cell morphology was related to cell activity and senescence (39). Cell morphology could be considered to be an indicator for cell properties.

A previous study found that administration of human MSC-derived EVs reduced the brain levels of *IL-1*β, but not *IL-6*, in a dose-dependent manner (9). We found that canine MSCs-EVs derived from SFM 2 reduced inflammation, mainly *IL-1*β, but not *TNF-*α and *IL-6*, by microglial cells in response to LPS stimulation. These findings are similar to those in studies using rat or human MSCs. On the other hand, human embryonic stem cell-derived EVs downregulated the expression of proinflammatory cytokine genes, including *IL-1*β, *TNF-*α and *IL-6* by human monocyte cell lines in response to LPS stimulation (40). Another report found that EVs derived from stem cells of human exfoliated deciduous teeth reduced production of *TNF-*α and *IL-6* by BV-2 cells in response to LPS stimulation (41). These differences could vary with species, cell origin, culture condition, and *in vitro* and the *in vivo* experimental system. In order to confirm the immunomodulatory effect of MSC-derived EVs, the signaling pathways for the production of pro-inflammatory cytokine needed to be investigated in the future studies.

We collected EVs derived from canine MSCs cultured in SFM conditions, because FBS contains significant quantities of EVs derived from bovine serum (42). There are few reports of canine MSC-derived EVs derived from normal medium containing FBS (43). Contamination of xeno-protein may increase the risk of immune reaction and transmission of infectious agents. In addition, FBS-derived EVs affects the immune response of macrophages to LPS (44).

EVs can be collected by ultracentrifugation, filtration, polymer precipitation, ion exchange chromatography and size-exclusion chromatography (9, 45). Ultracentrifuging gave greater enrichment in EV marker proteins, despite harvesting of considerably less protein than was precipitated (46). Isolation methods, as well as the culture conditions, could influence the yield and the functional characteristics of the EV sample obtained. In the present study, MSC-EVs were collected by ultracentrifuging, but this method is not suitable for large-scale production of EVs. It has been estimated that milligrams of EVs may be needed to treat dogs in clinical trials. Size exclusion chromatography has recently been found to efficiently isolates EVs with immunosuppressive activity (47). There is a lack of good manufacturing practice guidelines for large-scale manufacturing MSC-derived products. Further studies are needed to manufacture MSC-EVs on the large scale.

In conclusion, canine MSCs were found to survive and secrete EVs under the SFM condition. These EVs can reduce the inflammation and be useful for the clinical applications of canine MSC-derived EVs. These results warrant further studies of the use of SFM for producing MSCs-derived EVs.

## Data Availability Statement

The raw data supporting the conclusions of this article will be made available by the authors, without undue reservation.

## Ethics Statement

The animal study was reviewed and approved by the Experimental Animal Committee of Osaka Prefecture University.

## Author Contributions

YK and KY participated in study design, all data collection and analysis, and manuscript preparation. HN participated in funding, study design, all data collection and analysis, troubleshooting problems during the study, manuscript preparation and editing, and provided laboratory facilities and equipment. HK and SM participated in study design and manuscript preparation and provided laboratory facilities and equipment. KT participated in technical assistance and manuscript editing. NF and RN participated in funding, study design and manuscript editing. J-iJ and YT participated in study design, technical assistance and manuscript editing and provided laboratory facilities and equipment. HA participated in study design, manuscript editing, and provided laboratory facilities and equipment. All authors approved the final manuscript.

## Conflict of Interest

The authors declare that the research was conducted in the absence of any commercial or financial relationships that could be construed as a potential conflict of interest.
